# Molecular and neurocircuitry mechanisms of social avoidance

**DOI:** 10.1007/s00018-020-03649-x

**Published:** 2020-09-30

**Authors:** Anne-Kathrin Gellner, Jella Voelter, Ulrike Schmidt, Eva Carolina Beins, Valentin Stein, Alexandra Philipsen, René Hurlemann

**Affiliations:** 1 Division of Medical Psychology, Department of Psychiatry, University Hospital, Venusberg-Campus 1, 53127 Bonn, Germany; 2 Department of Psychiatry and Psychotherapy, University Hospital Bonn, Venusberg-Campus 1, 53127 Bonn, Germany; 3 Department of Psychiatry, School of Medicine and Health Sciences, University of Oldenburg, Hermann-Ehlers-Str. 7, 26160 Bad Zwischenahn, Germany; 4 Research Center Neurosensory Science, University of Oldenburg, 26129 Oldenburg, Germany; 5 Department of Psychiatry Und Psychotherapy, University of Göttingen, Von-Siebold-Str. 5, 37075 Göttingen, Germany; 6 Institute of Human Genetics, University Hospital Bonn, Venusberg-Campus 1, 53127 Bonn, Germany; 7 Institute of Physiology II, University Hospital Bonn, 53115 Bonn, Germany

**Keywords:** Social avoidance, Social anxiety, Neuronal circuits, Modulators of behavior, Translational models

## Abstract

Humans and animals live in social relationships shaped by actions of approach and avoidance. Both are crucial for normal physical and mental development, survival, and well-being. Active withdrawal from social interaction is often induced by the perception of threat or unpleasant social experience and relies on adaptive mechanisms within neuronal networks associated with social behavior. In case of confrontation with overly strong or persistent stressors and/or dispositions of the affected individual, maladaptive processes in the neuronal circuitries and its associated transmitters and modulators lead to pathological social avoidance. This review focuses on active, fear-driven social avoidance, affected circuits within the mesocorticolimbic system and associated regions and a selection of molecular modulators that promise translational potential. A comprehensive review of human research in this field is followed by a reflection on animal studies that offer a broader and often more detailed range of analytical methodologies. Finally, we take a critical look at challenges that could be addressed in future translational research on fear-driven social avoidance.

## Introduction

From an evolutionary perspective, the social environment is not only crucial for survival and reproductive success but substantially shapes physical and mental health [1]. Under certain circumstances and in response to environmental cues, social species including humans can deviate from their social nature and avoid social contact. Such behavior includes healthy as well as pathological manifestations. In animals, social avoidance can function as a survival technique or is part of submissive behavior, often provoked by social threat, e.g. an intruding dominant conspecific. In humans, social avoidance is often expressed as a behavioral symptom of anxiety, aiming at the avoidance of feared social evaluation and related negative emotions. Many psychiatric disorders are characterized by prominent interpersonal problems, including exaggerated social avoidance and aberrant control of approach-avoidance actions. Therefore, translational efforts have been made to better understand the molecular and neural network mechanisms underlying social approach-avoidance behavior to combat diseases that are marked by social avoidance. In this regard, animal research is instrumental in deciphering the molecular and neuronal underpinnings of social avoidance and for identifying new targets for potential neurobehavioral and pharmacological treatments. In the present paper, we present an overview of the molecular and neurocircuitry mechanisms underlying social avoidance. First, we focus on experimental work in humans and afterward on animal models. Hereby, we cover a broad spectrum from research on healthy to pathological social avoidance, including neural mechanisms and the influence of various modulators on social avoidance behavior. We conclude this review by discussing challenges and suggesting improvements for translational research on social avoidance in future studies.

### Social avoidance in the healthy human model

Social avoidance is a physiological and sometimes even life-saving facet of human behavior that is accompanied by aversive emotions and thoughts upon expecting or experiencing social situations. It can therefore function as a safety behavior that prevents or alleviates feelings of shame and anxiety [2]. In experimental settings, social avoidance is mainly assessed as an instinctive, automatic response to social threat, which is fear-driven or part of subordinate behavior. Within this framework, avoidant responses are modulated by subjects’ level of anxiety and by previous episodes of social stress. To examine the sub-clinical range of social avoidance, questionnaires are used to classify healthy subjects into high-anxious vs. low-anxious. Social avoidance is often not assessed separately but as part of self-report questionnaires targeting social anxiety, e.g. the Liebowitz Social Anxiety Scale (LSAS-SR) [3]. However, self-assessments are prone to distortions, e.g. as a consequence of subjective self-awareness, along with a tendency to select socially accepted answers. Given the aforementioned limitations, the focus here lies on the behavioral probes of social avoidance, some of which can also be used for neuroimaging studies, including functional MRI (fMRI). Several paradigms have been devised to map social avoidance as well as its opposite, approach behavior. Among these, the Approach-Avoidance Task (AAT) is one of the most frequently used. Specifically, participants approach positive social stimuli (happy faces) or avoid negative social ones (angry or fearful faces) by pushing a button or moving a joystick towards or away from themselves. Often, the AAT is subdivided into two conditions: an affect-congruent condition, in which participants approach positive stimuli and avoid negative stimuli, and an affect-incongruent condition requiring the opposite, i.e. to approach negative stimuli and avoid positive stimuli [4–6]. This subdivision makes it possible to also assess the control of social approach-avoidance behavior which can be dysfunctional in psychiatric disorders [6]. Affect-congruent responses result in faster reaction times than affect-incongruent ones, described as the congruency effect [6, 7]. High-anxious participants however tend to avoid both happy and angry faces, most likely because any of these express some form of feared social interaction [8, 9]. While the AAT tests basic approach-avoidance tendencies, these tendencies may be more pronounced under conditions of socially evaluative stress in which compensatory mechanisms become insufficient.

To investigate the interference of socially evaluative stress with approach-avoidance, the Trier Social Stress Test (TSST), a laboratory stress experiment usually performed outside the fMRI scanner, is used in combination with other tasks such as the AAT to create a naturalistic, stressful and socially-threatening situation [5, 10]. The TSST consists of a mock job interview, including a free speech and the performance of mental arithmetic tasks in front of an application committee, where committee members constantly maintain a neutral facial expression [11–13]. A significant increase in cortisol levels has confirmed the TSST’s ability to provoke psychosocial stress [13, 14]. To examine the direct effects of increased psychosocial stress inside the fMRI scanner as done by Lederbogen et al. [15], the Montreal Imaging Stress Task (MIST) was developed. Similarly to the TSST, the MIST consists of a computer-based mental arithmetic task and includes a social evaluative threat component [16].

There have been various experimental attempts to quantify the magnitude of social avoidance. One such study was performed by Schultz et al. [17]. Specifically, the authors devised an fMRI choice task, where participants had to choose between a risky and a safe option to win money. When choosing the safe option, participants received a predetermined fixed amount of money. The outcome of the risky option, which resulted in a game of dice against a human partner and therefore in social interaction, varied between either zero or three euros. The authors measured how often participants engaged in an uncertain social interaction as opposed to choosing the safe option of making money. At the behavioral level, the task thus allows to estimate the monetary costs that social avoidance comes within social contexts. On the neural level, the task maps decision-making related processes and responses to social feedback as a function of subjects’ level of anxiety.

Other methods established to examine social avoidance in humans include go/no-go and social incentive delay tasks, often operationalized by implementing social or monetary rewards and the avoidance of punishment [18–20]. Harari-Dahan and Bernstein [21] utilized the Key-Presses Task [22], which allows participants to shorten or extend the viewing time of a given stimulus, thereby enabling subsequent examination of avoidance behavior. In a synopsis of the animal to human translational paradigms relevant for approach-avoidance conflict decision making, Kirlic, Young, and Aupperle [23] highlight two classes of tasks that probe social approach-avoidance conflicts: (1) social trust games involving monetary incentives by which levels of trust and cooperation with other players are assessed and (2) eye gaze tasks, given that the attentive focus on other facial expressions is crucial for enabling social interactions. Less fixation on the eyes as well as gaze aversions are distinctive features of social avoidance and social anxiety disorders [2, 24–27].

### Neural mechanisms of social avoidance in humans

Consistent with the premise that social avoidance is characterized by a fear-driven propensity to reduce feelings of anxiety and shame in social contexts [2], the amygdala has been identified as one of the core regions involved in social avoidance in humans. Several studies report an amygdala hyperactivation in response to socially threatening cues, e.g. fearful and angry faces [20, 28, 29]. Exaggerated amygdala reactivity is also associated with a greater preferred distance to such stimuli [30]. Amygdala reactivity varies according to subjects’ level of anxiety, with high-anxious and high-avoidant participants exhibiting greater amygdala responses, especially when facing social feedback or decisions of whether or not to engage in social interaction [17, 20, 31]. Interestingly, similarly exaggerated amygdala activity towards socially threatening stimuli has been modeled after the injection of endotoxins, aimed at causing an inflammatory response in healthy subjects. The resultant feelings of disconnection have been interpreted as reflecting sickness-induced social withdrawal [32], which therefore is another potential source of avoidance behavior in humans. Increased amygdala activation has also been associated with delayed responses during a forced approach to fearful faces, confirming the involvement of the amygdala in orchestrating fear-induced social avoidance [18]. Normally the amygdala does not act in isolation but is tightly top-down controlled by the anterior prefrontal cortex (aPFC) [33]. Consistent with this model, Kaldewaij et al. [4] conclude that voluntary regulation of approach-avoidance behavior is marked by aPFC-induced top-down inhibition of the amygdala. With the advent of better tasks and high-resolution fMRI, specific roles have been attributed to subdivisions of the aPFC. First, the lateral frontal pole (FPI) has been implicated in regulating action tendencies subserving the control of approach-avoidance behavior via the ventral amygdalofugal bundle [34]. Second, affect-incongruent responses during the AAT evoke increased activity in the left lateral orbitofrontal cortex (OFC), underlining the importance of this subregion in control of approach-avoidance behavior [6]. Socially anxious subjects not only display an increased amygdala response to negative social stimuli, but also decreased activity in the nucleus accumbens (NAc) while receiving positive feedback from a social partner [17], suggesting that the mesolimbic reward circuit is dysfunctional in social avoidance. Possibly related to this are increased striatal responses to angry vs. happy faces in go/no-go tasks, as has been documented in high-avoidant participants [20]. Furthermore, increased ventral striatum/NAc activation was triggered by the anticipation of both avoiding social punishment or receiving a social gratification [19], suggesting that social avoidance can be highly rewarding and thereby stimulate a gain of illness.

**Fig.1:**
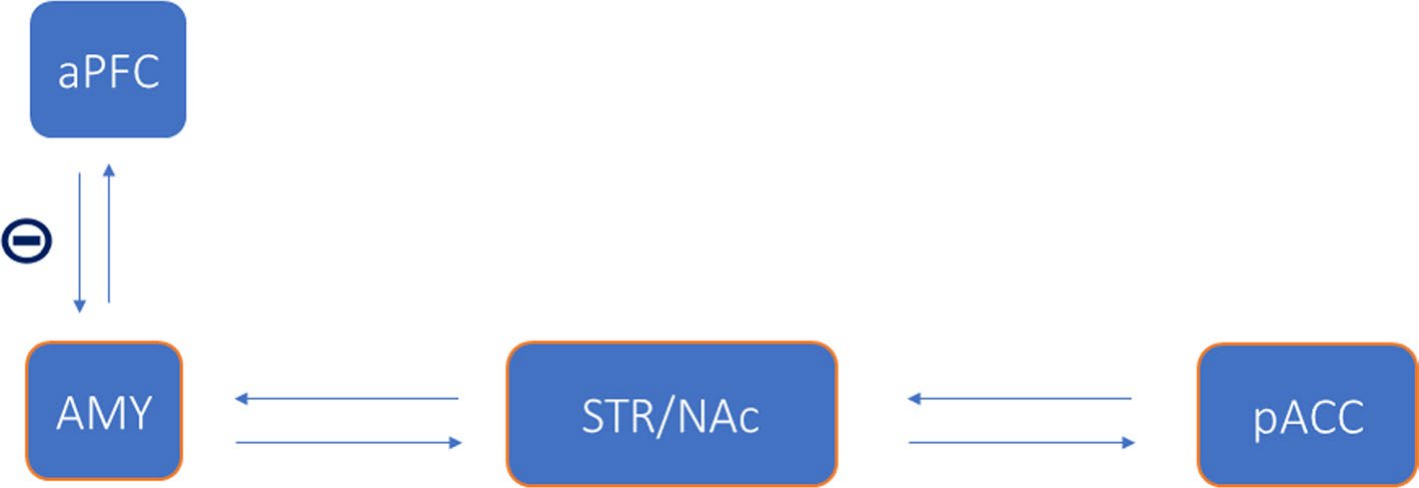
Neurocircuits associated with social avoidance in the healthy human model. Based on findings that: (1) The aPFC regulates approach-avoidance actions by top-down inhibition of the amygdala. (2) The amygdala has repeatedly been found to be hyperactive in response to social threat, specifically in socially anxious and avoidant participants. (3) The mesolimbic reward circuit is possibly altered in socially anxious participants as indicated by reduced NAc activity in response to social reward. (4) The functional connectivity between the amygdala and NAc, as well as NAc and pACC, alters as a function of social anxiety. *aPFC* anterior prefrontal cortex, *AMY* amygdala, *STR* striatum, *NAc* nucleus accumbens, *pACC* perigenual anterior cingulate cortex

Since the amygdala and NAc both possess important roles in the neural processing of social avoidance, functional connectivity between the two may be relevant. Indeed, during decisions whether or not to engage in social interactions, higher levels of anxiety were found to be associated with increased functional connectivity between the right NAc and the amygdala, accompanied by decreased functional connectivity between the right NAc and the perigenual anterior cingulate cortex (pACC) [17]. Chang and Yu additionally highlight the importance of the thalamo-hippocampal-insular/midbrain circuit, where resting-state effective connectivity changed in response to the TSST [35]. Overall, there is limited evidence regarding the underlying neural substrates of social avoidance in healthy humans. Most studies investigating the neural and behavioral underpinnings of social avoidance in healthy subjects have employed the AAT in various versions and in combination with the TSST. Of note, the AAT rather reflects automatic approach-avoidance tendencies than an actual decision process. Meanwhile, more recent approaches in the research of social avoidance have emerged, including go/no-go and other decision-making tasks. The majority of studies apply emotional facial expressions as experimental triggers of social avoidance responses, given that these stimuli represent salient carriers of nonverbal social information. Neuroimaging results emphasize the importance of amygdala, striatum/NAc, and their interactions, as key neural substrates of social avoidance, with top-down control of approach-avoidance actions being mediated by anterior prefrontal regions (summarized in Fig. 1). Thus, the neural underpinnings of social avoidance resemble those implicated in general avoidance behavior [36–38] and emotional processing (for a summary see [4]).

Overall, the evidence largely confirms the vigilance-avoidance hypothesis, which describes an enhanced vigilance towards social threat in socially anxious subjects, followed by avoidance behavior [39]. High-avoidant participants additionally display an impassiveness to social reward [40]. Furthermore, the response patterns measured in fMRI studies involving social reward and punishment [17, 19] often resemble those found with non-social incentives tasks [41, 42]. Such overlap has already been observed by Lin et al. [43]. Further research is needed to distinguish precisely between non-social and social avoidance to clarify ambiguous results. More recent advances in fMRI study design include a shift from static to dynamic approaches (e.g. virtual reality paradigms or video recordings), which might be beneficial for creating a more natural setting [44–46]. Despite technical hurdles, hyperscanning methods capturing two-person dyads are also becoming more relevant [44, 47].

### Pathological social avoidance in humans

If its intensity and/or frequency exceeds a certain level, social avoidance develops into pathological forms that can lead to complete social isolation, inability to work, and significant suffering. Social avoidance ranges on a stepless spectrum from functional-adaptive to inflexible-maladaptive. In principle, two forms of pathological social avoidance can be discriminated against, namely social anxiety with and without paranoid symptoms. The latter are mainly characterized by a biased perception of reality (attribution bias), that leads to irrational mistrust and suspicion of others and thus often also to hostile beliefs and rejection hypersensitivity [48].

### Social anxiety disorder (SAD)

Pathological social avoidance can occur, at least transiently, in almost any psychiatric disorder except acute mania and is, furthermore, the main characteristic of a social anxiety disorder (SAD) which is also called social phobia, e.g. in the ICD-10 diagnosis catalog. Besides SAD, social anxiety occurs very frequently in patients with an avoidant personality disorder. SAD is one of the most frequent mental illnesses [49] with an approximate lifetime prevalence rate of 12.1% [50]. There are several rule-outs to consider before diagnosing the symptom of social avoidance or psychiatric disorder SAD. Importantly, introversion and shyness are sometimes misconstrued as SAD, but are certainly within the normal limits of personality characteristics. Diagnosis criteria for SAD vary slightly between the ICD-10, DSM-5 and the upcoming ICD-11 diagnosis catalogs which define mental disorders according to phenotypic characteristics. In ICD-10, criteria for social phobia are met if an individual suffers from significant emotional distress either due to the non-psychotic fear of being in the center of attention or due to the fearful avoidance of social situations, given that he or she concomitantly considers these symptoms, including the characteristic fear to embarrass oneself in social situations, as excessive and unreasonable. In addition, two anxiety symptoms according to criterion B of the ICD-10 diagnosis agoraphobia need to be present together with either blushing, urgency or fear of micturition/defecation and/or fear of vomiting during the feared social situation. Finally, this symptom complex should be restricted to the feared situation and should not occur due to other disorders such as schizophrenia to fulfil the ICD-10 criteria of social phobia [51]. In ICD-11, among other changes, the social phobia has been termed SAD like in DSM-5 and, furthermore, a specification that symptoms must endure for at least some months has been added to its diagnostic criteria [52]. In contrast to ICD-10, DSM-5 illustratively summarizes general, psychological, and somatic anxiety symptoms occurring in SAD under the term “panic attack” and, furthermore, highlights differences in the clinical presentation of SAD between children and adults by stating that the duration of the SAD syndrome must persist at least 6 months in individuals under 18 years [53].

Criteria of psychiatric diagnoses such as SAD can be assessed in clinical expert interviews and by validated diagnosis-specific and/or transdiagnostic inventories. The widely used Structured Clinical Interview for DSM (SCID) has been proven to detect SAD with reasonably high specificity and sensitivity [49, 54]. Interestingly, some of the instruments assessing specifically the presence and intensity of social anxiety have been published long before the official recognition of SAD as a psychiatric illness in 1994 [55] e.g. the Social Avoidance and Distress Scale published in 1969 [56]. Today, the Liebowitz Social Anxiety Scale is one of the most used self-rating scales for social anxiety assessment [55]. In addition, there are a number of other valuable instruments for SAD assessment such as the Social Phobia Inventory, the Brief Social Phobia Scale and the Social Phobia and Anxiety Inventory [55].

### Other psychiatric conditions associated with social avoidance

Psychiatric disorders are generally highly comorbid with each other. It is well accepted that SAD is more frequent in female than in male subjects [57, 58]. Some researchers found sex differences in SAD comorbidity patterns. For instance, in the US National Comorbidity Survey-Replication sample, women with SAD were more likely to suffer from comorbid posttraumatic stress disorder (PTSD), specific phobia, generalized anxiety disorder (GAD), whereas men suffering from SAD were more likely to have conduct disorder and comorbid substance abuse [57]. However, these findings are not surprising as the latter two diseases are known to be principally more frequent in men and anxiety disorders and PTSD are generally more frequent in women. In a cohort from the German Mental Health Survey, 2% suffered from full DSM-IV SAD, 3% from symptomatic (i.e. one DSM-IV criterion missing) and 7.5% from subthreshold SAD. 87.8% of the subjects with full SAD suffered of at least one other mental disorder during the past 12-month period with 60% of them having three or more comorbid disorders and only 12.2% showing no comorbid condition with pure cases being younger than comorbid cases [58]. In this sample, all three SAD groups were associated with obsessive–compulsive disorder (OCD) as well as with anxiety disorders, particularly with GAD, panic disorder (PD), and agoraphobia [58].

Social evaluative concerns are the most common type of paranoid thoughts [59]. Paranoid ideas are usually accompanied by aversive emotions ranging from mild feelings of discomfort to intense distress. Social avoidance due to paranoid thoughts occurs frequently inter alia in paranoid personality disorder and, together with passive social withdrawal, also in schizophrenia [60]. In schizophrenic patients, passive social withdrawal, which occurs due to lack of motivation, is related more to negative symptoms while social avoidance is related more to positive symptoms and is an active behavior resulting from unwarranted fear, hostility or distrust [61].

### Treatment of social avoidance in neuropsychiatric disorders

Avoidance is a behavioral manifestation of fear and anxiety that tends to generalize particularly in individuals with an elevated vulnerability for mental disorders. PTSD patients avoid trauma-related cues, GAD patients avoid a constantly increasing amount of everyday risks, and phobic patients the object of phobia such as social situations in the case of SAD. Accordingly, cognitive-behavioral treatments (CBT) targeting pathological avoidance by gradual exposure to stimulating cues are effectively used in the treatment of SAD and other anxiety disorders [62, 63]. Patients suffering from these conditions also significantly profit from pharmacotherapy, in particular from treatment with serotonin reuptake inhibitor antidepressants such as paroxetine which has repeatedly been shown to act effectively against SAD in adult patients [64, 65]. Accordingly, paroxetine, sertraline, and the serotonin-noradrenaline reuptake inhibitor venlafaxine have been approved by the FDA for SAD treatment [66]. Furthermore, some experimental treatment approaches such as augmentation of exposure-based CBT with the partial NMDA receptor antagonist D-cycloserine [67] constitute promising potential novel treatment options for social anxiety. Schizophrenia-associated social avoidance was shown to improve significantly in response to treatment with antipsychotics [68, 69]. In summary, these facts point at divergencies in the neurobiological underpinnings of pathological social avoidance with and without paranoid and psychotic symptoms and stimulate the hypothesis that the latter might be associated rather with an impaired serotonergic transmission while social avoidance with paranoid and psychotic symptoms might possibly be related to excessive dopamine signaling.

### Neural mechanisms of pathological social avoidance

The high comorbidity of psychiatric disorders points at the imprecision of their definitions that calls for a novel biology-informed taxonomy. Accordingly, meta-analyses have shown that genetic alterations as well as changes in brain structure and function in psychiatry are often non-specific with similar or even identical changes appearing in distinct mental disorders [70]—the same accounts for neural, endocrine and molecular mechanisms in SAD patients. A very recent review aimed at untangling the functional network alterations of anxiety disorders and revealed that SAD patients show alterations in the functional connectivity (FC) of several frontal regions (i.e. the orbital cortex, the prefrontal cortex (PFC) and the ACC) with the limbic system (i.e. the hippocampus, the amygdala and the temporal lobe) while GAD patients are characterized by limbic-prefrontal and mesocorticolimbic FC changes and PD patients show limbic-PFC and frontoparietal FC alterations in emotional and network tasks, respectively, as well as ACC-amygdala FC changes [71]. A meta-analysis of functional neuroimaging studies performed during treatment of SAD concluded that psychotherapy and pharmacotherapy impact on different brain regions in adult SAD patients with the amygdala being influenced by neither treatment [72]. In contrast, a more recent review on neuroimaging amygdala response markers for SAD treatment found that responses of the amygdala decreased, and the FC of amygdala pathways changed in response to treatment of SAD [73]. In accordance, a just-published fMRI-study suggested a better long-term outcome of treatment to be associated with strengthened amygdala connectivity with regulatory regions while poorer treatment effects were paralleled by changes in sensorimotor and visual areas [74]. Several recent volumetric MRI studies suggested a role for the striatum, a critical component of reward and motor systems, and related constructs (e.g. intolerance of uncertainty) in SAD [70]. In detail, SAD has been found associated with an enlarged striatal volume [70] that, in turn, could be reduced, at least in two striatal subregions, by successful paroxetine treatment [70, 75]. However, findings on the striatal volume in SAD are not unequivocal [76] thus pointing to the necessity for additional studies with larger sample sizes [70] as well as to the importance of confounders which comprise comorbid disorders, age, and drug treatment [76]. In the latter meta-analysis that includes eleven studies on the grey matter volumes (GMVs) of different brain regions of SAD patients vs. healthy controls (HC), the GMV of the left putamen, which is a part of the dorsal striatum, was reduced in SAD while those of the supplementary motor area (involved in motor control), the middle occipital gyrus (involved inter alia in object recognition) and the left precuneus (involved inter alia in episodic memory, visuospatial processing, and reflections upon self) were found to be enlarged [76]. Another meta-analysis showed that the neural response within the face-processing system in SAD involves a complex network and is not limited to emotional structures as faces evoked a higher response in SAD not only in limbic regions such as in the bilateral amygdala but also in non-limbic structures such as the globus pallidus (implicated in motor control), the visual cortex, the superior temporal sulcus (implicated in social perception) and the PFC (involved in executive functions such as decision making) [77]. Despite the inconsistencies of the studies on the role of brain regions and networks in SAD, there is considerable evidence for a (non-exclusive) role of the limbic system, the sensorimotor and visual areas, and their networks in SAD pathobiology.

## Modulators of social avoidance in humans

### Glucocorticoids

Cortisol is the major effector hormone of the hypothalamic–pituitary–adrenal (HPA) axis, which, together with the sympathetic nervous system (SNS) forms the two main stress hormone systems in humans. Since both systems are essential for stress coping, they belong to the most studied biological systems in clinical psychiatry. Studies indicate augmented processing of social threat after cortisol administration, which may be specific to high-avoidant and high-anxious participants [78, 79]. Furthermore, high cortisol levels in response to the TSST are correlated with heightened avoidance of social threat in SAD patients [10] as well as diminished active approach-avoidance behavior [5]. The latest meta-analysis of cortisol reactivity in response to psychosocial stress in mental disorders revealed significant differences between male and female SAD patients as men, but not women exhibited an increased cortisol response to psychosocial stress. In addition, the authors found sex-dependent differences in HPA axis reactivity also among other mental disorders such as major depression and schizophrenia [80]. A study on the influence of psychosocial stress on the HPA axis reactivity in females suffering from PTSD identified two HPA axis reactivity subgroups with an almost identical expression of PTSD symptom severity but a difference in the intensity of trauma-related dissociative symptoms and of the prevalence of combined early life and adult traumatization [81]. The latter suggests that these endocrine differences might possibly result from well-known trauma-induced epigenetic shaping of HPA-axis related genes [82] which might occur in any (psychiatric) disorder and, thus, would be an interesting topic to study in SAD patients. In comparison to major depression, PD, and PTSD, the epigenetic regulation of the HPA axis seems to be markedly understudied in SAD.

### Testosterone

Social approach-avoidance actions are influenced by different modulators of mood and behavior. For instance, single-dose administration of testosterone promotes an increased approach to social threat, accompanied by decreased social avoidance, in healthy controls [83, 84] as well as in SAD patients [24, 85]. Accordingly, SAD-associated social avoidance behavior has been linked to low endogenous testosterone levels [86], indicating a dysregulation of the hypothalamic-pituitary–gonadal (HPG) axis with possible effects on SAD-relevant brain networks [87]. Furthermore, a recent clinical study showed that endogenous testosterone levels in females are predictive of the success of exposure therapy in SAD patients with low baseline and high reactive pre-treatment testosterone levels being related to larger post-treatment reductions in symptom severity [86]. The testosterone-induced bias away from avoidance towards the approach of social threat may result from the influence of testosterone on the amygdala, prefrontal cortex, and their interactions [88–90]. At the social-behavioral level, these findings are likely reflected by the established role of testosterone in enhancing social dominance to defend social rank [89] – an effect which is regulated by cortisol [91].

### Oxytocin

A neuropeptide hormone, whose anxiolytic [92, 93] and prosocial effects in humans [94, 95] are well established, is oxytocin (OXT). Oxytocin has been associated with a variety of mental disorders such as major depression, schizophrenia, anxiety disorders [96], PTSD [97], and also with SAD [98, 99] and was, furthermore, found to be associated with emotional neglect in children [100]. Endogenous peripheral oxytocin levels have been found reduced in SAD patients during a trust game [101]. A genetic study associated genetic variants of the oxytocin gene with adolescent social anxiety symptoms and an epigenetic study revealed that, in SAD patients, reduced methylation of the oxytocin receptor (OXT-R) gene was related to increased symptom severity and to an elevated HPA axis response to social stress [99]. Nasal administration of oxytocin has been found to increase the social approach and decrease avoidance [21, 102–104], most likely by its inhibition of the amygdala [93, 105, 106]. It further counteracted SAD-associated alterations in the functional connectivity of various brain regions [96, 107, 108]. Table 1 summarizes studies on oxytocin treatment, among them one treatment study which revealed that oxytocin treatment did not reduce SAD symptom severity, although it at least resulted in several other beneficial therapeutic effects [109] and two fMRI studies found oxytocin to increase the connectivity of the amygdala with various other regions [107]. Two other studies showed an influence of oxytocin treatment on reward behavior [98, 110]. Finally, another research group found that oxytocin application reduced the activities of both the amygdala and the PFC [106, 111].

**Table 1:** Studies on oxytocin treatment in social anxiety disorder (SAD) patients

First author	Amount and use of placebo	Study design/experimental paradigm	n	Results
Dohdia et al. 2014	24 IU, nasal spray vs. placebo	Resting-state fMRI	18 SAD vs. 18 CON	In SAD patients, OXT enhanced resting-state functional connectivity of the left and right amygdala with several cortical areas thereby reversing the suppressed amygdala-frontal connectivity observed relative to CON in response to placebo
Fang et al. 2017	24 IU, nasal spray	reward motivation task that assessed willingness to work for self vs. other monetary rewards	52 SAD	No main effect of OXT, but OXT-treated less socially anxious individuals who received OXT worked harder for other vs. own rewards, compared to high socially anxious individuals
Gorka et al. 2015	24 IU, nasal spray vs. placebo	Assessment of fear-related amygdala reactivity with fMRI	17 SAD vs. 18 CON	In SAD patients, but not in CON, OXT enhanced functional connectivity between the amygdala and the bilateral insula and middle cingulate/dorsal anterior cingulate gyrus during fear processing
Guastella et al. 2009	24 IU, nasal spray vs. placebo	OXT was applied before 4 of in total 5 exposure treatment sessions	25	Mental representation of self but not SAD symptoms, life-impairment measures or dysfunctional cognition improved in OXT -treated group
Hurlemann et al. 2019	24 IU, nasal spray vs. placebo	Temporal discounting task and reward evaluation	33 SAD vs. 37 CON	OXT increased the patient choices (later-larger) across all participants. SAD patients showed more impulsive preferences under placebo compared to CON while this difference disappeared after OXT treatment
Labuschagne et al. 2010	24 IU, nasal spray vs. placebo	Assessment of fear-related amygdala reactivity with fMRI	18 SAD vs. 18 CON	OXT had no effect on amygdala activity to emotional faces in the CON group, but attenuated the heightened amygdala reactivity to fearful faces in SAD patients
Labuschagne et al. 2012	24 IU, nasal spray vs. placebo	Assessment of fear-related cortical reactivity with fMRI	18 SAD vs. 18 CON	OXT reduced heightened activity to sad faces in the medial prefrontal cortex extending into anterior cingulate cortex in SAD patients to levels similar to those of controls

*RCT* randomized controlled trial, *fMRI* functional magnetic resonance, *CON* controls, *OXT* oxytocin

While oxytocin-induced approach behavior in female subjects, at least in positive social contexts [112], pair-bonded male volunteers avoided attractive women, an effect which was absent in single men and may facilitate long-term relationships [113]. Given that the effects of oxytocin on social approach and avoidance are a person—and context-dependent, rigorous study designs are required to account for this complexity in future research.

### Dopamine and serotonin

Oxytocin and testosterone both interact with the monoaminergic neurotransmitter systems including the dopaminergic pathways [114], which may be dysfunctional in social avoidance as indicated by the aforementioned alterations in the mesolimbic circuit. Indeed, SAD patients display both reduced density of dopamine uptake sites [115] and lower-than-normal D2-receptor binding potential in the striatum [116]. Concentrating on extrastriatal brain regions, SAD symptom reduction correlates with increased D2 receptor binding in medial prefrontal and hippocampal regions [117], however, overall prefrontal D2 receptor levels were found to be elevated [118]. Although the evidence is limited and inconsistent, SAD may be characterized by altered dopaminergic neurotransmission. Together with dopamine, the serotonergic (5-HT) system is one of the key neurotransmitter pathways underlying social-emotional processing. An increase in serotonin levels mediated by selective serotonin reuptake inhibitors (SSRIs) or supplementation of the precursor tryptophan, is associated with positively biased processing of social stimuli [119–121]. Conversely, decreased serotonin levels after tryptophan depletion have been revealed to cause a shift towards the increased perception of negative social stimuli, perhaps mediated by increased amygdala-prefrontal connectivity [122]. Tryptophan depletion further increases the autonomic stress response to social evaluation in SAD patients treated with SSRIs [123]. In addition, SAD patients display reduced serotonin-1A receptor (5-HT1A) binding in limbic and paralimbic regions, most significantly in the amygdala, anterior cingulate cortex, insula, and dorsal raphe nuclei [124]. There has been growing interest in genetic research to identify variations in the serotonergic system underlying altered social-emotional processing, with mounting evidence indicating the importance of a polymorphism in the promoter region (5-HTTLPR) of the human serotonin transporter gene (SLC6A4). Subjects carrying the 5-HTTLPR short allele display increased levels of anxiety and an increased amygdala response to social threat [125, 126] together with higher levels of social avoidance [127], compared to subjects with copies of the long allele. In contrast, Stein et al. [128] failed to demonstrate a genetic linkage between generalized social phobia and the serotonin transporter (5-HTT) or 5HT2A receptor genes. A recent positron emission tomography (PET)-study highlighted the importance of both, dopamine and serotonin, in SAD by reporting an association of SAD with an increased expression of dopamine and serotonin transporters in reward- and fear-associated brain regions [129].

### Brain-derived neurotrophic factor (BDNF)

Excessive stress-induced signaling of glucocorticoid hormones such as cortisol inhibits BDNF synthesis and thereby reduces neural plasticity. The upregulation of BDNF through pharmacotherapy may reverse brain atrophy and thereby contribute to the antidepressant effect in stress-related disorders [130]. The BDNF single nucleotide polymorphism (SNP) Val66Met has been linked to increased attention towards the social threat, together with elevated amygdala-prefrontal connectivity as well as exaggerated amygdala and hippocampal responses to social cues in anxious and depressed patients [131, 132]. However, relative to animal models discussed in the second part of this review, data on the human BDNF polymorphism Val66Met and its relevance for social stress and avoidance is harder to interpret (see for review [133]). Different studies indicate both higher social anxiety scores in Met-carriers of both sexes [134], whereas males also showed attenuated perception of social stress and consecutive HPA axis response compared to male homozygous Val carriers [135], an effect found to be opposite in females [136]. Overall, human research on BDNF and social avoidance has received less attention despite being a valuable translational target for understanding neuroplasticity-related effects.

### Endocannabinoids

The endocannabinoid system (ECS) is a widely expressed neuromodulatory system involved in the regulation of various physiological and pathological processes. It is comprised of cannabinoid receptors CB1 and CB2, their endogenous ligands (endocannabinoids), and enzymes involved in endocannabinoid metabolism [137]. In the CNS, endocannabinoid signaling acts as a retrograde feedback mechanism at both excitatory and inhibitory synapses. Strong depolarization of postsynaptic neurons induces synthesis of the endocannabinoid 2-Arachidonoylglycerol (2-AG) that activates presynaptic CB1 receptors, which in turn inhibits further neurotransmitter release [138]. The ECS thus provides important homeostatic control over the neuronal activity. Components of the ECS are expressed in other CNS cells as well, such as astrocytes and microglia, thus enabling communication between cell types. Furthermore, the ECS has well-documented functions in peripheral tissues, including the regulation of immunological and metabolic processes [139, 140]. Importantly, CB1 receptors are highly expressed in brain regions associated with the regulation of emotion, cognition, and stress-responses [141] and disturbances in endocannabinoid signaling have been implicated in a range of psychiatric disorders in both clinical and preclinical studies including social anxiety [142]. Strong evidence for the ECS’ involvement in mental health came from observations on the anorectic drug rimonabant (SR141716), a selective cannabinoid receptor type 1 (CB1) inverse agonist [143], which caused serious mood alterations and depressive symptoms in up to 10% of patients, leading to a worldwide withdrawal of its approval in 2008 [144, 145]. Furthermore, there is some evidence linking genetic variation in the ECS to phenotypes related to social and emotional processing. In healthy subjects, SNPs in the CNR1 gene have been associated with differences in gaze duration and brain responses towards happy faces presented during fMRI studies [146, 147]. CB1 signaling might thus play a role in the perception of certain emotional/social signals. Furthermore, variants in ECS genes have been associated with several relevant personality traits or psychiatric disorders, including high neuroticism and low agreeableness [148], bipolar disorder [149], and panic disorder [150]. In recent years, there has been a lot of interest in the therapeutic potential of cannabinoid substances, especially the phytocannabinoid cannabidiol (CBD) (reviewed by [151]). A few small clinical studies have demonstrated anxiolytic effects of CBD in patients with SAD or avoidant personality disorder, using anxiety questionnaires and/or simulated public speaking tests [152–154]. Functionally, the anxiolytic action of CBD was related to altered activity in limbic and paralimbic brain areas, assessed by regional cerebral blood flow [153].

In sum, it is the complex interplay of various modulators that influences social avoidance and guides behavior. Further research, especially in clinical populations, is mandatory. An overview of the aforementioned modulators and their influence on social approach-avoidance behavior is shown in Table 2.

**Table 2:** Modulators of social avoidance in humans

Modulator	Findings
Testosterone	*Increase* Promotes social approach and decreases avoidance of social threat *Decrease* Low endogenous testosterone levels are linked to social avoidance in SAD *Other* Endogenous testosterone levels are predictive for therapy success in female SAD patients
Cortisol	*Increase* Enhances processing of social threat, specific for anxious/avoidant participants Correlates with increased social avoidance of social threat in SAD patients Correlates with diminished active approach-avoidance behavior Cortisol responses to psychosocial stress are elevated in male SAD patients
BDNF	*Polymorphism linked to* Increased attention towards social threat Exaggerated amygdala and hippocampal responses to social cues in psychiatric patients Higher social anxiety scores Increased cortisol responses to social stress in females but attenuated responses in males
Oxytocin	*Increase* Increases social approach and decreases avoidance in a highly context-dependent manner Counteracts SAD-associated alterations in the functional connectivity of various brain regions Does not reduce SAD symptom severity but results in other beneficial therapeutic effects *Decrease* Endogenous peripheral oxytocin levels are reduced in SAD patients during a trust game *Other* Reduced oxytocin receptor methylation is related to: an increased SAD symptom severity an elevated HPA axis response to social stress
Dopamine	Extrastriatal D2 receptor levels are elevated in SAD patients Increased D2 receptor binding in prefrontal and hippocampal regions correlates with SAD symptom reduction Reduced striatal density of dopamine uptake sites and lower D2 receptor binding potential in SAD patients Increased expression of dopamine transporters in reward- and fear-associated brain regions in SAD patients
5-HT	*Increase* Induces a positively biased processing of social stimuli *Decrease* Increases perception of negative social stimuli Increases stress response to social evaluation in SAD *Other* Reduced 5-HT receptor binding in limbic and paralimbic regions in SAD patients Increased expression of serotonin transporters in reward- and fear-associated brain regions in SAD patients Polymorphism of the 5-HT transporter gene linked to: Increased levels of anxiety and social avoidance Increased amygdala response to social threat

### Investigation of social avoidance in animals

Defining normal social behavior in animals, including its affiliative and aversive poles, depends on multiple factors like species, sex, age, and the environment they live in (i.e., laboratory vs. wildlife). Social behavior comprises and requires approach and avoidance, interaction, recognition, and formation of memories and can be driven by social reward or fear. In rodents and other gregarious species, fear of new social interaction partners is usually overcome by the physiological drive to socially approach and interact. These opposing processes are regulated via different brain circuitries and neurotransmitters and -modulators [155, 156]. We want to reflect on the structural and molecular mechanisms of social avoidance and withdrawal in the context of animal models and stressors, which are linked to the aforementioned human behavior and neuropsychiatric disorders.

Social avoidance in animals is evoked by social adversity or threats, such as social isolation or instability, maternal separation or abuse, social fear conditioning, subordination, and social defeat [157]. Systemic injection of lipopolysaccharides (LPS), bacterial endotoxins eliciting a strong inflammatory response, transiently (up to 12 h) also induces social withdrawal as part of sickness behavior [158] and can be attenuated by anti-inflammatory drugs like minocycline that reduce cytokine expression in the hippocampus [159]. Nevertheless, social stressors are far more common in research on social avoidance and fear. Especially social defeat models, based on the resident-intruder paradigm, offer acute (1 day) or chronic (up to 30d) time frames to elicit different sets and durations of symptoms [160, 161] and bear high translational value [162]. Assessment of avoidance comprises variations of confronting the test animal with a shielded or freely accessible familiar or unfamiliar, same- or opposite-sex, younger or dominant counterpart and the choice to interact or not [157, 163]. Interpretation of the different tests depends on the preceding behavioral model or treatment, sex, age, and species under investigation and have to be chosen carefully as altered social interaction can occur independently or in combination with other symptoms linked to a depressive or non-socially anxious phenotype [157, 160, 164].

### Neuronal networks underlying social avoidance in animals

The circuitry determining social behavior involves the anterior and ventromedial hypothalamus, amygdala (AMY), the bed nucleus of the stria terminalis (BNST), lateral septum, periaqueductal gray (PAG) among others. It connects with the dopaminergic mesocorticolimbic motivation and reward network (ventral tegmental area (VTA), nucleus accumbens (NAc), and prefrontal cortex (PFC)) [155, 165].

The VTA serves as a dopaminergic hub modulating activity in the NAc and the medial prefrontal cortex (mPFC) and neuronal activity patterns in the VTA correlate with social interaction and avoidance [166–168]. Neuronal hyperactivity induced by a hyperpolarization-activated cation channel–mediated current (*I*_*h*_) was detected in the VTA of avoidant mice after social defeat stress [169, 170] and acute upregulation of *I*_*h*_ currents by a single infusion of the antiepileptic drug and mood stabilizer lamotrigine similarly enhanced social avoidance [171]. Conversely, upregulation of voltage-gated potassium channels (VGKCs) Kcnf1, Kcnh3, Kcnq3 is triggered by even higher *I*_*h*_currents observed in non-avoidant mice and resulted in neuronal hypoexcitability in the VTA. Inline, local induction of hypoexcitability in the VTA by either genetic upregulating of potassium channels [169] or pharmacological potentiation of *I*_*h*_ current via infusion of lamotrigine for 5 days [171] restored normal social behavior. Neuronal properties within mesocorticolimbic projections were found to be discriminatory of avoidant vs. non-avoidant phenotypes after social defeat stress with an activity increase in mesolimbic VTA-NAc projections whereas decreased firing rates were observed in the VTA-mPFC connection [167, 172, 173]. The relevance of these distinct pathways in the context of stress-induced social avoidance was demonstrated when phasic, not tonic, optogenetic stimulation was capable to elicit social avoidance in live animals by either activation of the VTA-NAc [167, 172, 173] or inhibition of the VTA-mPFC projections [167]. Moreover, inhibition of the VTA-NAc connection turned avoidant into non-avoidant phenotypes [167]. Together, these findings underline how circuit-specific alterations of excitability underly social interaction and its disruption. Mechanistically, chronic but not acute treatment with the antidepressants fluoxetine and imipramine restored normal social behavior in avoidant mice, which—for fluoxetine—was accompanied by normalized excitatory properties of VTA neurons as well as the reversal of gene expression changes in the NAc [170, 174]. Together, these findings demonstrate the importance of balanced neuronal activity in the VTA in social behavior. Golden et al. demonstrated, that besides altered functional plasticity described before, also structural plasticity is disrupted in brain regions modulating social behavior: they discovered a reduction of the small Rho GTPase Rac1, which is involved in synaptic remodeling, in the NAc of avoidant mice. Normal social behavior was in turn restored together with neuronal structural plasticity by constitutively inducing Rac1 activity [175].

Within the circuitry of VTA, NAc and mPFC, activation of projections from the infralimbic part of the PFC to the NAc reversed social avoidance in mice [176]. In line with this, the activation state of the mPFC is relevant for the formation of social avoidance after psychosocial stress. After exposure to chronic social defeat stress (CSDS), reduced expression of the immediate-early genes Zif268 and Arc indicated decreased neuronal activity in the mPFC specifically in those mice that developed social avoidance [177] and this avoidant phenotype could be abolished by optogenetic stimulation of neuronal activity the mPFC during social interaction [177]. Similarly, the downregulation of Zif268 expression in the mPFC was shown to induce social anxiety [178]. Inline, upregulation of delta-FosB, a transcription factor enhancing inhibitory neurotransmission mediated by cholecystokinin (CCK) between interneurons and pyramidal cells [179], in the mPFC was only found in socially avoidant animals [176]. Stress duration impacts not only the set of symptoms but also gene expression patterns in the PFC. In a study by Bondar et al., 10d of CSDS sufficed to induce social avoidance in mice when confronted with an aggressor in the absence of other behavioral changes associated with depression. This was accompanied by a high level of gene regulation in the PFC and upregulation of pathways related to the formation of extracellular matrix and cell adhesion that can eventually alter cortical neuroplasticity. After 30d of continuous defeat stress, a more severe phenotype emerged with generalized social avoidance and symptoms of depression whereas most of the initially regulated genes of the 10d group had returned to control levels. These adaptions could be linked to epigenetic mechanisms as chromatin remodeling histone methyltransferases were significantly enriched and downregulated in the depressive 30d group vs. the 10d stress group. In contrast, a distinctively different, smaller set of genes, mostly related to neuroplasticity like spine dynamics and neurotransmission, was found to be downregulated in the PFC of depressive, generally socially avoidant mice [180]. This points us towards highly adaptive processes during prolonged periods of stress that include reversal of early effects already concomitant with signs of social dysfunction. Eventually, this mounted in a complex avoidant phenotype associated with dynamic, plastic neuronal networks. Slower neuronal conduction in the PFC might also be a relevant mechanism for developing social avoidance. Hypomyelination and myelin remodeling occurred in socially avoidant mice after CSDS or chronic social isolation [181, 182]. Induction of demyelination in the mPFC led to social withdrawal and both spontaneous remyelination [182] and pharmacological enhancement of myelination [181] normalized social behavior. The authors hypothesized a defect of the differentiation of oligodendrocyte progenitor cells induced by chronic social defeat stress [182]. In line with this, mice deficient for the myelin paranode protein CNP1 show social withdrawal at baseline [183] and are more susceptible to stress-induced avoidance [184].

The PFC is directly connected to other brain regions controlling anxiety and fear responses and dysfunction of these networks reflect a pathophysiological mechanism underlying social avoidance. Disrupted feedback control between PFC, AMY and VTA is suggested as an important cause for CSDS-induced avoidance in mice [185, 186]. In hamsters, avoidant behavior after the conditioned social defeat was associated with a lack of activation in projections from the mPFC to the amygdala [187]. The mPFC also normally exerts top-down control over parts of the brainstem (periaqueductal gray, PAG) to modulate social approach and avoidance and disruption of this connection was found to induce social avoidance [188]. Activation of the PAG together with context-specific circuits of the ventromedial hypothalamus was also detected in the face of social threats [189, 190].

Volumetric magnetic resonance imaging (MRI) studies in rodents also tried to detect specific anatomic alterations in avoidant phenotypes. In a post hoc analysis, the size of the hypothalamus was increased, while that of the bed nucleus of stria terminals (BNST), component of the extended amygdala [191], and dorsal raphe nuclei (DRN) in the brainstem was decreased [192]. While the BNST has recently been reviewed extensively as a mediator of social salience in aversive social contexts via oxytocin [193], the DRN serves as the major brain-wide source of serotonin (5HT) but also specifically promotes activation of the VTA-NAc pathway via serotonin and glutamate signaling [194]. There is a general influence of social stimuli on hippocampal function and neurogenesis proven in adult mammals [195] although the role of aversive stimuli is less clear. Hippocampal alterations found with avoidance behavior also depend on the age in which it is provoked. In juvenile mice, social stress-induced avoidance correlated with chronically suppressed hippocampal neurogenesis [196] that is required for forming normal social behavior [197]. In adult rodents, persistent avoidant behavior correlated with chronically increased hippocampal neurogenesis, possibly as a compensatory mechanism after initially suppressed neurogenesis [198]. Data on volumetric changes in adult avoidant animals after chronic psychosocial stress is not consistent, showing either arrested growth [199] or increased volumina [192] of hippocampi. Interestingly, a pre/post MRI study revealed higher hippocampal volume as a predisposition for developing social withdrawal after chronic social stress [199]. The ventral hippocampus (vHPC) serves as information storage for memorizing and evaluating social stimuli [200]. It sends glutamatergic inputs to the NAc that specifically determine susceptibility for social avoidance, as enhancement of this transmission clearly promoted avoidant behavior [201]. The amygdala and its subdivisions are widely connected [202] with other brain regions relevant for social behavior, including the circuitries formed with the mPFC [176] described above, but also with the hippocampus. These connections modulate social behavior and fear, as activation of the basolateral amygdala (BLA) in primates [203] and specifically of the BLA-vHC circuitry in rodents [204] reduced social interaction in a variety of social settings.

Neuronal networks adapt at different time scales to changing tasks by modulating synaptic connections, often referred to as synaptic plasticity. It describes the ability of existing synapses to strengthen or weaken over time and functionally, these plastic changes are mediated by altering the number of neurotransmitter receptors at the post-synaptic site or the pre-synaptic release of neurotransmitters. The hippocampus is probably the best-studied brain region in synaptic plasticity, but its adaptions regarding social avoidance are less clear. Recently, it was demonstrated that CSDS animals with a socially avoidant phenotype show no hippocampal long-term potentiation (LTP) [205]. Unfortunately, this study did not additionally compare the non-avoidant phenotype emerging from chronic stressor. Yet, another study showed no difference in LTP after CSDS, but an increased long-term depression (LTD) mediated by the metabotropic glutamate receptor mGluR5 [206]. These two recent studies confirm the heterogenous picture; however, induced behavior of social avoidance in special or stress in more general clearly manifests in changes of synaptic function. The apparently contradictory findings in functional synaptic plasticity might reflect the complexity of the system. Especially the underlying electrophysiological methods rely on several parameters that can change the outcome. While LTP and LTD is classically studied in young adolescent animals (3 to 6 weeks of age), the induction of social avoidance is mostly based on older animals.

## Modulators of social avoidance in animals

### HPA axis and glucocorticoids

Under healthy conditions, a well-concerted oscillation of circulating glucocorticoid hormones is maintained by the HPA axis in both animals and humans. The hypothalamus stimulates the pituitary gland with corticotropin-releasing hormone/factor (CRH, CRF), thereby sending adrenocorticotropic hormone (ACTH) to the peripheral adrenal cortex, which in turn secretes cortisol in humans and corticosterone (CORT) in rodents into the bloodstream. These glucocorticoids exert their effects via the glucocorticoid receptor (GR), which is widely distributed in the brain and most other peripheral tissues [207, 208]. The HPA axis contains several feedback loops both within the brain and in the periphery. Within the brain, binding of glucocorticoids to the GR in the hippocampus or hypothalamus inhibits CRH synthesis, which eventually lowers circulating glucocorticoid levels. The HPA axis is a physiological system helping the organism to cope with physical and psychological stressors, influences social interaction [209], matures with aging and its dysregulation has been extensively studied in the face of neuropsychiatric disorders and in their preclinical models [210, 211].

Dysregulation of the HPA axis is not only found in impaired adult social responses but also at juvenile life stages in animals as well as in socially anxious children and adolescents [212]. Several stress models in animals effectively induce social avoidance in pre-adult age stages, in part with delayed onset of avoidant behavior [213] and often temporally dissociated from other affective symptoms found in adult rodents [196, 214, 215]. One marker of social avoidance in those juveniles is reduced HPA reactivity in response to social interaction [213, 215]. Interestingly, social withdrawal in juveniles that had been exposed to an abusive mother could be rescued via systemic application of corticosterone [216], whereas exogenous corticosterone in adults induced avoidance in previously unaffected animals after chronic social defeat [217]. Hence, HPA response to social interaction depends on age and context but ultimately influences social behavior.

Circulating corticosterone levels have been studied repeatedly in various social stress models but mostly in chronic social defeat variations that induce social avoidance. In general, the elevation of blood corticosterone is found both in socially avoidant and non-avoidant mice directly after classic CSDS and thus is unlikely the direct cause of the disrupted social interaction [169] but rather the complex downstream- and feedback mechanisms within GR-signaling. Social avoidance after CSDS is not only accompanied by elevated plasma CORT but also higher expression of CRH in the hypothalamus, demonstrated to rely on gene demethylation and decreased GR expression in the hypothalamus and in the hippocampus of avoidant animals [218, 219]. Systemic treatment with the antidepressant imipramine attenuated both avoidant behaviors together with Crf mRNA methylation levels indicating drug effects on the epigenetic level [219]. Systemic direct inhibition of the GR during CSDS prevented social avoidance in adult mice [196]. Antidepressants are thought to exert part of their effects via modulation of the GR [211]. Treatment of avoidant mice with the selective serotonin uptake inhibitor (SSRI) escitalopram restored social interaction, lowered plasma corticosterone, and restored GR protein expression in the hippocampus [218]. The flavonoid icariin, investigated for its antidepressant efficiency alleviated social avoidance and lowered hypothalamic CRH mRNA potentially via restoration of decreased GR levels in the hippocampus [220]. In the mPFC, regulation of mainly glucocorticoid-responsive genes was found in avoidant animals after 10 but not 30 days social stress, indicating a high relevance of GC-dependent mechanisms for the initiation of social avoidance but not primarily for its sustainment [180]. Alteration of HPA function appears to be at least in part independent of the specific social stressor applied to the individual: Socially deprived mice developed signs of social avoidance and HPA hyperreactivity (elevated plasma CORT and adrenal gland weight) [221] and undefeated rats housed with defeated cagemates also developed avoidance together with a hyperactive HPA axis [222], revealing the contagious quality of social stress and reinforcing translational value of animal models.

### Testosterone

Most animal research regarding social behavior is traditionally performed in males, neglecting the relevance of sex differences [223]. In most gregarious species used in the laboratory environment (mice, rats), adult males are more dominant, territorial, and easily demonstrate aggressive same-sex behavior in contrast to their female conspecifics which is mainly attributed to the dimorphism for the gonadal steroid hormone testosterone [224].

Data on testosterone in avoidance behavior of males is not clear-cut: Lowered testosterone levels were repeatedly found in submissive, fight-avoidant males ranging from rats [225] and hamsters [226] to rhesus monkeys [227] although others found them unchanged in subordinate hamsters [228] or rats [229]. In general, sex differences have been addressed in too few studies regarding social avoidance so far with a distinct exception for the “social hormone” oxytocin (see later section). Stack et al. found molecular changes in the frontal cortex of males but not females modulating social interaction and the authors hypothesized testosterone to normally prevent downregulation of neuronal activity in this brain region underlying social avoidance [178]. It was only recently shown in a non-social stress model how resilience in males was mediated by testosterone by lowering vHPC-NAc excitability [64]. As the same projection was found to regulate resiliency towards social stress [201], its sex-hormone dependent exploration has interesting implications for humans and should be considered in future studies.

### Oxytocin

Oxytocin (OXT) is a highly conserved neuropeptide found in humans and animals modulating social behavior [193, 230, 231]. In the previous chapters, mesocorticolimbic and associated networks and their alterations were already described as pivotal for the development of social avoidance. During rewarding social behavior, dopamine sent from the VTA to the NAc acts prosocial [168]. Upstream of the VTA and regulating its excitatory level, OXT neurons in the paraventricular nucleus (PVN) of the hypothalamus increase activity during social interaction and thus enhance sociability. In contrast, inhibition of OXT signaling in the VTA reduced social preference in mice and hamsters [232–234]. Downstream of the VTA, local inhibition or deletion of presynaptic OXT receptors (OXT-R) in the NAc abolished social preference. To add to the regulatory effects of OXT within different brain circuitries, serotonergic cells in the DRN that project to the NAc are also equipped with presynaptic OXT-R and their local inhibition abolished social preference [232]. These findings underline the role of intact local serotonin signaling for prevention of social avoidance, pointed out in the following section, in concert with the actions of OXT in mesolimbic areas. Although the amygdala also contains OXT-R and is involved in both social and anxiety behavior, local antagonism did not change baseline or stress-mediated disrupted social behavior [235]. In a model of male adolescent social instability, increased OXT-R density was found in the NAc and dorsolateral septum (dlS) [236] and systemic treatment with an OXT-R antagonist increased stress-induced social avoidance [237]. After social fear conditioning, OXT-R binding was also increased in the dorsolateral septum, amygdala and parts of the hippocampus and injection of OXT into the dlS restored social behavior [238]. In contrast to the previous findings, OXT-R overexpression in the dlS promoted avoidance of an aggressor after social defeat whereas local knockdown of the receptor prevented the formation of this aversive social behavior that relies of formation of social memories [239]. This demonstrates how the avoidance-modulating effects of endogenous and exogenous oxytocin depend not only on context but also the anatomic effector sites. Similar to the effects in the mesolimbic network, non-region specific intracerebroventricular (i.c.v.) infusion of an OXT-R antagonist or its systemic administration decreased social preference and interaction in rats and mice [232, 240, 241] and even evoked strong social avoidance in the latter [235]. In the same study, avoidance towards an aggressor could be prevented by i.c.v. administration of OXT even resulting in social preference towards the former defeater showing its prosocial potential. OXT effects do not only depend on the target region or application method but also sex. Social avoidance could be induced by local infusion of an OXT-R antagonist into the BNST in socially defeated adult female mice but not males [240, 241] and modulation of social behavior by OXT-R agonism and antagonism was demonstrated to be simultaneously dose- and sex-dependent in hamsters [242]. Intranasal OXT reduced social interaction in females in one study [240] but led to increased conditioned social preference in females but not males in another [243]. Different treatment and test protocols might, in part, also account for the different effects as a dose-dependency of systemically applied OXT for social behavior outcomes was clearly demonstrated in mice [244]. Taken together, the data on OXT-mediated modulation of social interaction in socially challenged or even stressed animals is mixed and seems to depend highly on context, age, or sex of the individual – congruent to the pool of data in humans.

### Dopamine and BDNF

With a focus on the mesocorticolimbic network, motivation for social behavior is promoted or inhibited by tuning the dopaminergic activity of the VTA and its projections, which is tightly connected to BDNF signaling, a strong regulator of neuronal plasticity [245]. As the hub of the social reward network, specifically the VTA-NAc (but not VTA-mPFC) pathway acts pro-social via D1-receptor signaling in the NAc [168]. In acute and chronic stress paradigms, avoidant behavior is mediated by both increased dopamine [173, 246] and BDNF signaling [172] within the NAc, yet social withdrawal after chronic stressors seem to rely strongly on BDNF [173]. Interestingly, activation of dopaminergic signaling in the NAc in naïve California mice induced social avoidance in females but not males whereas defeat-induced avoidance could be reversed by dopamine antagonism in the NAc only in males [246]. Enhanced dopaminergic signaling via D2 but not D1 receptors within the NAc was revealed to facilitate avoidant behavior [247]. Hyperactive dopaminergic VTA neurons induce the upregulation of BDNF and its downstream cascades (i.e., PI3K/Akt, ERK1/2, Gsk-3ß) [169, 174] and Gadd45b, a mediator of DNA methylation [248]. In line with this, inhibition of ERK signaling [169] or Gadd45b function [248] normalized avoidant behavior. Direct, local deletion of the BDNF gene in the VTA, as well as local inhibition of the BDNF receptor Tropomyosin receptor kinase B (TrkB) in the NAc prior to social stress, prevents the development of avoidance [172–174]. Conversely, bilateral BDNF infusion into the NAc enhanced susceptibility for social avoidance [169]. Together, this is demonstrating BDNF- and experience-dependent plasticity necessary for abnormal social behavior. The NAc response to VTA activation is locally regulated via the hypothalamic-borne neuropeptide CRH. Local antagonism of the CRH receptors in the NAc prevented the rise of BDNF-levels and thus social avoidance [172]. Of note, systemic TrkB agonism reduced whereas antagonism promoted social avoidance in defeated mice [249]. In line with this, mice with decreased BDNF signaling via dysfunctional TrkB receptor showed intensified and prolonged social avoidance [250]. This points at the translational challenge of how acute vs. chronic, local vs. global alteration of BDNF-TrkB signaling affects affective behavior [251, 252] due to the various effector sites and circuits of BDNF action. For instance, BDNF levels were found reduced in frontal cortex, hippocampus, and hypothalamus of avoidant animals [221]. In line with this, increased hippocampal BDNF signaling is capable of preventing social withdrawal whereas its local inhibition promotes it [253, 254]. The SNP Val66Met, which reduces activity-dependent neuronal BDNF release in humans [255] and in transgenic mice [256], protected mice from the development of social avoidance after CSDS. Surprisingly, excitability of VTA neurons was not different compared to wildtypes, but NAc of the homozygous Val66Met mice contained 50% less BDNF, underlining its local relevance for regulating social interaction [169].

As described in detail in the previous sections, hyperactive VTA-NAc dopaminergic projections are a key feature underlying avoidance behavior after psychosocial stressors. In contrast, mesocortical VTA-mPFC projections are hypoactive [167] with a consecutively marked reduction of dopamine turnover in the frontal cortex [257, 258] and DRN. Lowered frontal dopaminergic signaling appears to be specific for the socially avoidant phenotype since it was not altered in a chronic stress model that induced general anxiety but no social withdrawal [257]. Moreover, an acute defeat protocol that did not induce social avoidance led to increased prefrontal dopamine as a possible protective factor, whereas chronic defeat inhibited dopaminergic signaling in the mPFC via prostaglandins and thus enabled the development of social avoidance [258]. The dopamine receptor D1 was found downregulated in the mPFC of avoidant mice, and its local knockdown also promoted avoidance behavior [259]. The amygdala receives mesolimbic dopaminergic inputs that regulate social behavior. In prairie voles, increased dopamine signaling within the amygdala via the D1 receptors (but not D2 subtype) was shown to mediate social avoidance in response to repeated social defeat but interestingly also in stress-naïve voles [260]. Hence, the role of dopaminergic signaling in social avoidance is highly dependent on localization within the circuitry.

**Fig. 2:**
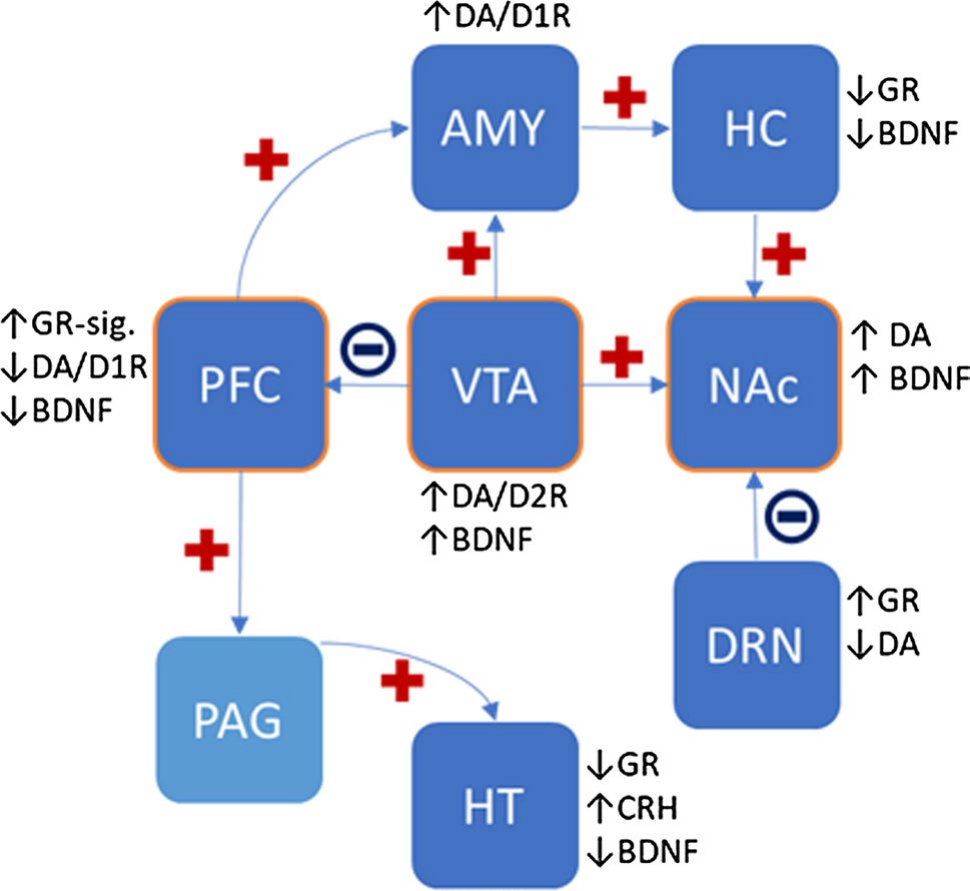
Alterations of circuitry and main findings of glucocorticoid/dopamine/BDNF signaling in socially avoidant animals. The mesocorticolimbic system consisting of VTA, PFC, and NAc is in the center of regulating stress-induced social avoidance in preclinical models. Projections with either activity-increase (plus) or -decrease (minus) determine the level of transmitter-release and expression of other modulators and their receptors in the target region (position of arrowhead). *BDNF *brain-derived neurotrophic factor, *CRH *corticotropin-releasing hormone, *D1R and D2R *dopamine receptor 1 and 2, *DA *dopamine, *GR *glucocorticoid receptor)

The actions of the circuitry eventually leading to socially avoidant behavior are in large parts connected by glucocorticoid, BDNF, and dopamine signaling and are summarized in Fig. 2.

### Serotonin

The serotonergic system with its main source in neurons of the dorsal raphe nuclei (DRN) is a modulator of socio-affective, in part GR-driven response independent of the classic HPA axis feedback loop. Treatment of socially avoidant mice with imipramine not only restored social behavior [174] but also downregulated HDAC6, a cytoplasmic lysine deacetylase. HDAC6 modulates hormone- and stress-induced GR translocation, in the dorsal raphe nucleus (DRN). Deletion of HDAC6 in serotonergic neurons of the DRN has been demonstrated to exert antidepressant, anxiolytic effects [261], prevented stress-induced social avoidance together with linked specific morphological alterations and hypoexcitability of the serotonergic cells and translocation of the GR despite elevated plasma CORT [217]. This underlines the importance of raphe circuit homeostasis which ultimately regulates the serotonergic tone of the brain. Serotonergic neurons of the DRN are also linked to the endogenous opioid system in regulating social avoidance. Inhibition of the p38alphaMAPK pathway in serotonergic neurons activated via the dynorphin/kappa opioid receptor (KOR) efficiently blocked the development of social avoidance [262]. The serotonergic tone of the brain, concerted via the DRN and regulated via the serotonin transporter (5-HTT), also modulates vulnerability towards stress-induced social avoidance. A 50% reduction of 5-HTT expression increased vulnerability without alteration of plasma corticosterone but lowered serotonin turnover in the frontal cortex [263]. In contrast, rodents and primates with a higher baseline concentration of 5HT in brain and plasma showed higher vulnerability to developing social avoidance in a socially crowded environment but failed to produce elevated CORT levels in response to the social stress found in non-avoidant conspecifics [264]. In line with this, acute treatment with the serotonin reuptake inhibitor (SSRI) Citalopram, which facilitates 5-HT transmission in the brain, has been shown to decrease social interaction in rodents [265]. In addition, transiently suppressed 5-HT1A receptor expression in the DNR during early postnatal development which disinhibits serotonergic neurons, leads to higher vulnerability to early life stress and reduced sociability in later life stages in general [266]. This shows, how balanced 5HT metabolism and signaling is a prerequisite for coping with (stressful) social situations and prevention of social avoidance.

### Endocannabinoids

The ECS and especially the CB1 receptor regulate behavior and stress-responses in rodents [142]. Mice genetically deficient for the CB1 receptor (Cnr1^−/−^) show phenotypes similar to clinical symptoms of anxiety and depressive disorders [267] and show certain differences in social behavior. For example, Cnr1^−/−^ mice show increased aggressive behavior when confronted with an intruder in their home cage [268, 269], while showing decreased social interaction in a novel environment [269], suggesting context- and stress-dependent regulation of social behavior by CB1 signaling. These findings are supported by a study demonstrating increased arousal/anxiety-like behavior in male Cnr1^−/−^ mice when exposed to a novel conspecific, which possibly contributes to enhanced social memory/discrimination in these mice [270].

CB1 signaling particularly affects social relationships between mothers and their offspring. Cnr1-/- dams show deficits in maternal care (pup retrieval), which is likely caused by their increased anxiety state and correlated with reduced oxytocin receptor and BDNF expression in the hippocampus [271]. Many of the behavioral phenotypes of Cnr1-/- mice, including increased anxiety and maternal care deficits, are also observed in mice deficient for DAGLa, the main enzyme responsible for 2-AG production in the CNS. These mice, however, did not show a phenotype in a social preference test [272].

Another aspect of social behavior regulated by endocannabinoid signaling is adolescent social play, which is critical for developing social competence in most mammalian species and shows clear sex-differences [273]. Social play behavior is a highly rewarding activity and the ECS is well known for modulating or interacting with neural systems involved in natural rewards, such as the opioid and dopaminergic system [274]. Generally, increasing endocannabinoid tone (e.g. by inhibiting endocannabinoid-degrading enzymes) increases social play behavior in rats, while blocking CB1 receptors reduces it [275–279]. It was further demonstrated that the masculinisation of social play (i.e. increased frequency and duration of play fighting) in juvenile rats is critically dependent on CB1/CB2 signaling [280]. Here, the treatment of neonatal rats with CB1/CB2 agonists masculinized social play behavior in female rats, while antagonist treatment feminized social play in male rats. Mechanistically, the endocannabinoid-mediated masculinization of social play behavior is possibly related to altered neuron-glia interaction in the amygdala [281]. In perinatal male rats, androgens cause an increase in endocannabinoid tone in the developing amygdala, promoting phagocytosis of newborn cells by microglia. This reduces the number of astrocytes that survive into adolescence, which in turn increases neuronal excitation during social play in male rats.

Next to the amygdala, other brain regions implicated in endocannabinoid effects on social behavior are the ventral hippocampus (vHPC), NAc, and VTA. In mice, social avoidance can be induced by optogenetic activation of BLA-NAc glutamatergic circuits, which could be prevented by systemically increasing 2-AG levels [282]. Furthermore, activation of CB1 receptors in the vHPC was shown to increase the firing of DA neurons in the VTA and neurons in the NAc shell, thereby disrupting normal social behavior and social recognition [283, 284]. These findings demonstrate region- and circuit-specific effects of endocannabinoid signaling on social behavior.

Finally, the ECS is critically involved in regulating stress responses and several studies have demonstrated a role for endocannabinoid signaling in the effects of stress on social behavior. In a model of PTSD, pre-treatment with cannabinoid agonists or FAAH inhibitors could prevent deficits in social recognition memory, as well as anxiety- and depressive-like behavior induced by a shocking reminder [285]. Similarly, inhibiting 2-AG degradation during SDS could prevent the development of social avoidance [286]. Oppositely, mice that lack DAGLa or CB1 receptors constitutively or on specific neuronal subpopulations are especially sensitive to the behavioral consequences of social stress [287, 288].

A summary of the modulators and their actions in social avoidance (induction or prevention) of animals is shown in Table 3.

**Table 3:** Modulators of social avoidance in animals

Modulator	Regulation pro social avoidance	Regulation contra social avoidance
Glucocorticoids and HPA axis	↓CORT (juvenile), specific ↑CORT (adult), non-specific ↑CRH in HT ↓GR in HT and HPC ↑GR-signaling in mPFC Systemic CORT application (adult)	Systemic CORT application (juvenile) Systemic inhibition of the GR (adult) Imipramine: ↓CRH in HT Escitalopram: ↓CORT, ↑GR in HPC Icariin: ↓CRH in HT, ↑GR in HPC
Testosterone	↓ testosterone (males only)?	↓ vHPC-NAc activity via testosterone?
BDNF	↑ BDNF in NAc and/or VTA ↓ BDNF in FC, HPC, HT Local infusion of BDNF in NAc Increase of BDNF-signaling in HPC Systemic TrkB antagonism	↓ BDNF in NAc and/or VTA Inhibition of TrkB signaling in NAc and/or VTA Inhibition of CRH-receptors in NAc by prevention of BDNF-increase Increase of BDNF-signaling in HPC Systemic TrkB agonism SNP Val66Met with ↓BDNF in NAc
Dopamine	↑ in VTA and NAc (via D2 receptors) ↓ in mPFC and DRN ↓ D1 receptor in mPFC ↑ DA-signaling via D1 in AMY knock-out of D1 receptor in mPFC	DA antagonism in NAc (males)
5-HT	↑5-HT in brain and plasma 50% reduced expression of 5-HTT ↓5HT in the frontal cortex ↓5-HT1A receptor expression and disinhibition of 5-HT neurons (P14-30) Citalopram: ↑5-HT transmission	Imipramine: restores properties of 5-HT neurons
Oxytocin	↑ OXT-R density in NAc and dLS ↑ OXT-R binding in AMY, HPC, dLS OXT-R overexpression in dLS Inhibition of OXT signaling in VTA Inhibition/deletion of OXT-R in NAc Inhibition of OXT-R in DNR OXT-R antagonist in BNST (females) Intranasal OXT (females) i.c.v. OXT-R antagonist Systemic OXT-R antagonist	Local injection of OXT into dLS Knock-out of OXT-R in dLS i.c.v. OXT Intranasal OXT (females)
Endocannabinoids	Activation of CB1 in HPC Inhibition of CB1 signaling knock-out of DAGLa knock-out -OUT of CB1	Systemic increase in 2-AG Inhibition of 2-AG degradation

### Challenges for future translational research on social avoidance

In the previous sections, social avoidance has been described and reflected from the viewpoints of human and animal studies as a primarily active withdrawal from social interaction, which can be a physiological, adaptive, and protective reaction to social threat and unpleasant social experience. However, social avoidance is not always fear-driven, but can also result from a decreased need for social interactions and thus be a passive trait [40] which was not focused in this review. Hence, a comparison of these two sides of social avoidance and their mechanisms might also be of interest in the future in understanding their role in disease and how to treat them.

The borderline between an active adaptive or maladaptive response is stepless and even more difficult to define when comparing human and animal behavior. Humans can be subjected to both multimodal self-reporting and investigator-based assessments regarding their behavior, underlying emotions, and cognitions whereas assessment in the most common studies of animal species, rodents, relies on careful observation and measuring. Theory of mind, the ability to take the perspective of others, is a key prerequisite for human social cognition although a recent review summons aspects of a theory of mind also in non-human species like apes, dogs, and some birds [289]. Data on social perspective taking or metacognition in rodents are scarce but has been reported in rats [290, 291]. The settings in which social behavior can be probed determines its translational value in both directions. Social stressors resembling acute and chronic physical and psychological abuse and helplessness in different life stages can be modeled in animals and often bear striking resemblance to human negative social experience. Nevertheless, there are also gaps between species that remain harder to bridge as, for example, humans can be subjected to and fear socially evaluative threats and express embarrassment. Similarities in behavioral outcomes were apparent in the grade of generalization of avoidance towards social stimuli (i.e., both happy and angry faces in humans and aggressive and non-aggressive conspecifics in rodents). Despite the higher cortical complexity in humans, similar circuitry for social cognition and impairment has repeatedly been described in rodents with emphasis on oxytocin and relatives [292, 293]. In depth knowledge of circuits and molecular mechanisms relies mostly on the cutting-edge techniques applicable in rodents and can not be simply transferred to other species for technical and ethical reasons. Hence, further exploration of cognitive psychology in rodents in regard to the systems covered in this review and beyond could serve as a valuable additional information source for neurobiological and -physiological research.

Focusing on circuitry and molecular regulation underlying social avoidance, we found high overlap between species in the mesolimbic dopaminergic system, the limbic regions and the prefrontal cortex. This comes with little surprise as the so called social brain and its components have repeatedly been described as highly conserved in mammals both anatomically and functionally [165, 294]. The differences or uncertainties in translating findings “from mouse to man” have been described as hurdles in pinpointing the causes for certain behavior and how to manipulate it. Nevertheless, the modulators of social avoidance covered and suggested in our review – from the more obvious glucocorticoids and oxytocin to the less frequently described endocannabinoid system within this context – offer exciting diagnostic and therapeutic levers. Advances in human and animal (f)MRI techniques but also high-resolution optical imaging in even freely behaving rodents offer analyses in more naturalistic settings. Biological sampling surely will remain difficult in comparison between species for example regarding brain tissue but translational options should be examined closer: cerebrospinal fluid is more easily attainable, even repeatedly and can be thoroughly analysed from small amounts due to high-throughput mass-spectrometry together with the more commonly sampled plasma or serum. There are still several challenges lying ahead to understand how effects on brain structure and function can be measured optimally and comparably in both humans and animals.
